# Statistical Analyses Support Power Law Distributions Found in Neuronal Avalanches

**DOI:** 10.1371/journal.pone.0019779

**Published:** 2011-05-26

**Authors:** Andreas Klaus, Shan Yu, Dietmar Plenz

**Affiliations:** 1 Section on Critical Brain Dynamics, National Institute of Mental Health, Bethesda, Maryland, United States of America; 2 Nobel Institute for Neurophysiology, Department of Neuroscience, Karolinska Institute, Stockholm, Sweden; 3 Stockholm Brain Institute, Stockholm, Sweden; University of Michigan, United States of America

## Abstract

The size distribution of neuronal avalanches in cortical networks has been reported to follow a power law distribution with exponent close to −1.5, which is a reflection of long-range spatial correlations in spontaneous neuronal activity. However, identifying power law scaling in empirical data can be difficult and sometimes controversial. In the present study, we tested the power law hypothesis for neuronal avalanches by using more stringent statistical analyses. In particular, we performed the following steps: (i) analysis of finite-size scaling to identify scale-free dynamics in neuronal avalanches, (ii) model parameter estimation to determine the specific exponent of the power law, and (iii) comparison of the power law to alternative model distributions. Consistent with critical state dynamics, avalanche size distributions exhibited robust scaling behavior in which the maximum avalanche size was limited only by the spatial extent of sampling (“finite size” effect). This scale-free dynamics suggests the power law as a model for the distribution of avalanche sizes. Using both the Kolmogorov-Smirnov statistic and a maximum likelihood approach, we found the slope to be close to −1.5, which is in line with previous reports. Finally, the power law model for neuronal avalanches was compared to the exponential and to various heavy-tail distributions based on the Kolmogorov-Smirnov distance and by using a log-likelihood ratio test. Both the power law distribution without and with exponential cut-off provided significantly better fits to the cluster size distributions in neuronal avalanches than the exponential, the lognormal and the gamma distribution. In summary, our findings strongly support the power law scaling in neuronal avalanches, providing further evidence for critical state dynamics in superficial layers of cortex.

## Introduction

**Figure 1:**
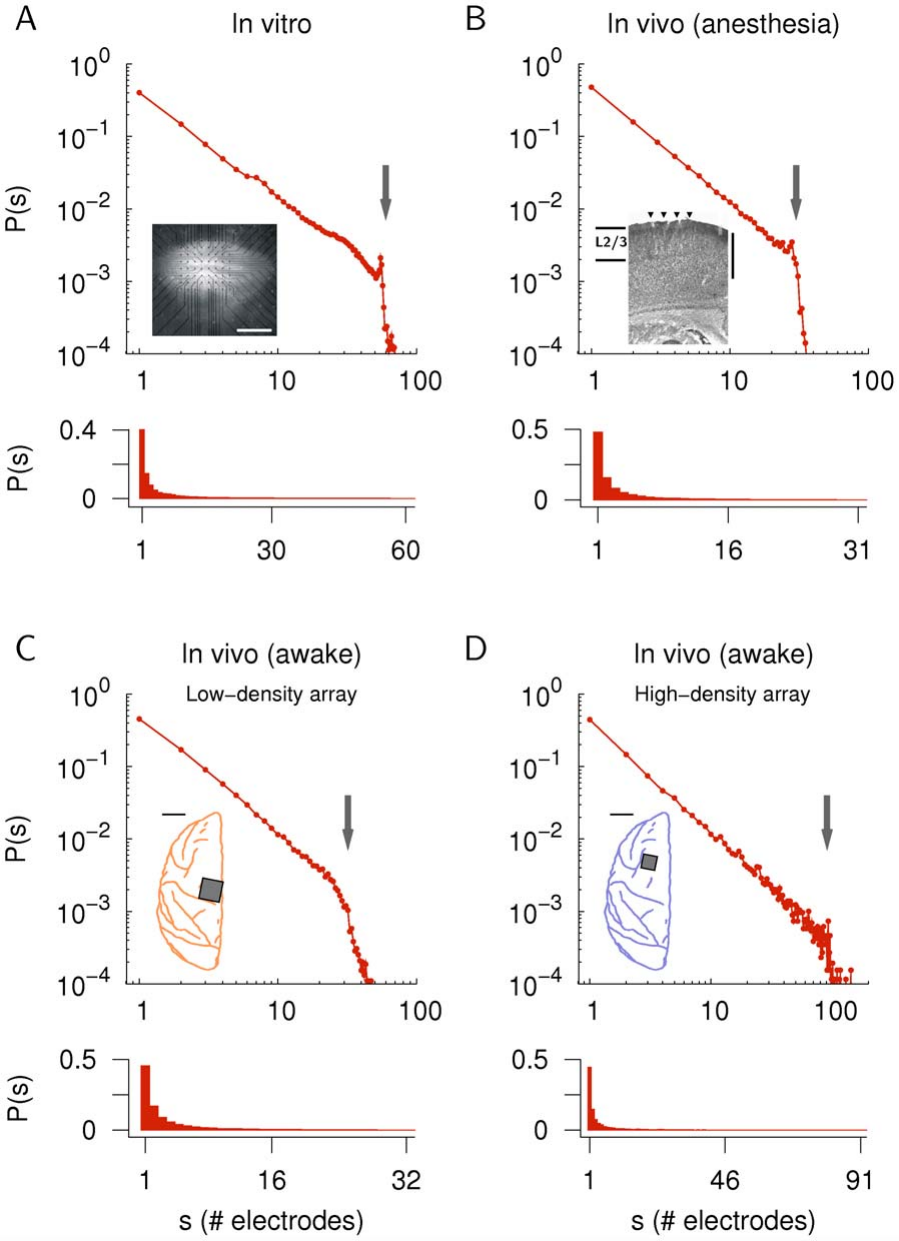
Avalanche size distributions analyzed in the present study. A. Average in vitro cluster size distribution in organotypic cortex slice cultures (60 electrodes, 7 cultures, *n* = 53,443 avalanches on average) in double-logarithmic (upper panel) and linear scale (lower panel). The data set was taken from [2]. Inset: view of a culture on a 8
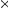
8 electrode array (scale bar, 1 mm). B. Average in vivo cluster size distribution from rat somatosensory cortex under urethane anesthesia (27–31 electrodes, 7 recordings, *n* = 22,321 avalanches on average). Data was taken from [4]. Inset: view of the insertion sites for an 8
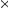
4 array (triangles) in cortical layer 2/3 (vertical scale bar, 1 mm). C. 43-min recording for monkey X (low-density microelectrode array with 32 electrodes in the left primary motor cortex, *n* = 45,574 avalanches). Data was taken from [5]. D. 30-min recording for the second monkey (monkey Y, high-density microelectrode array with 91 electrodes in the left premotor cortex, *n* = 24,877 avalanches). Insets in C and D show the location of the multielectrode arrays (scale bar, 10 mm). The size of the arrays (dark squares) is not shown in the actual scale. The number of electrodes in the individual arrays is indicated by arrows in the log-log plots (A–D).

Complex systems, when poised at the transition between order and disorder, exhibit scale-free dynamics [1]. These dynamics are characterized by a probability distribution of event sizes that follows a power law with exponent 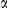:
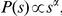
where *P*(*s*) denotes the probability of an event of size *s*. Recently, the size of neuronal activity cascades in superficial layers of cortex, measured by the number of negative threshold crossings of the local field potential (nLFP), has been suggested to be distributed according to a power law with exponent 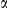 close to −1.5 [2]–[5] (Figure 1). This activity was termed “neuronal avalanches.” The exponent of −1.5 indicates that neuronal avalanches reflect long-range spatial and temporal correlations in the network as expected from critical dynamics [2], [6]–[8]. Accordingly, pharmacological manipulations that perturb communication beween neurons rapidly destroy the power law [2]–[4]. Similarly, spatial and temporal shuffling of recorded neuronal activities, which destroy such correlations and serve as randomized controls, also abolish the power law and instead result in a size distribution from an exponential family [4], [5], [9]. The range of sizes of neuronal avalanches has consistently spanned 1.5 to 3 orders of magnitude [2]–[5], and the cut-off of their distributions was shown to systematically change with system size, consistent with the hypothesis that the system is in a critical state [2], [5]. Importantly, the propagation of activity within neuronal avalanches is highly balanced, that is, one neuronal active site in the network on average spawns activity at one other site in the near future. Thus, avalanche dynamics fulfill the theoretical predictions for critical branching processes that exhibit both a power law in cascade size distributions with a slope of −1.5 and a critical branching parameter equal to unity [2], [10].

In many cases, deciding whether a given empirical distribution follows a power law and to determine its slope can be technically challenging [11]–[15]. Until recently, whether a given distribution is appropriately described by a power law was largely determined by visual inspection of the distribution in a double-logarithmic plot [2], [16], [17]. In such a presentation, a power law conveniently takes on the form of a straight line. For neuronal avalanches, this feature and the large deviation between the original distribution and shuffled, exponentially distributed controls were interpreted as evidence in favor of a power law [4], [5]. As pointed out previously [13], [14], [18], [19], such a visual approach suffers from a lack of statistical rigor in identifying a significant difference between a power law and an exponential or other alternative model distributions. This problem is worsened when the availability of the data is limited, for example, for small sample sizes or when the range of values over which the distribution is analyzed is narrow [13], [15].

The reasons described above emphasize the need for more stringent methods in testing the power law hypothesis for neuronal avalanches. The present study was aimed to provide such an analysis. More specifically, we performed the following three steps: (i) finite-size scaling analysis to motivate the power law model as an appropriate description for the distribution of avalanche sizes, (ii) parameter estimation of the statistical models to determine the slope of the power law and to allow the subsequent model comparison, and (iii) comparison of the power law and the exponentially truncated power law to the alternatives of an exponential, a lognormal and a gamma distribution. We re-examined multielectrode data on neuronal avalanches in different systems with various preparations, including organotypic cortex slice cultures in vitro [2], in vivo under anesthesia [4], and in vivo in the awake macaque monkey [5] (Figures 1A–C, respectively). In addition, we analyzed new data on ongoing activity in an awake macaque monkey recorded with a high-density array (91 channels, Figure 1D). The model comparison was done by a likelihood ratio test [14], [18], [20] and, additionally, by using a comparison that was based on the Kolmogorov-Smirnov (KS) statistic. Both tests clearly favored the power law models over the alternative distributions for all data sets. Furthermore, good fits were also obtained by the inverse Gaussian distribution, which describes a power law with fixed exponent −1.5 and additional cut-off function. Taken together, these results indicate that cluster size distributions in neuronal avalanches scale according to a power law with particular exponent 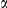 close to −1.5, which provides strong support for critical state dynamics in superficial layers of cortex.

## Results

In the present study, we analyzed the power law scaling of neuronal avalanches recorded from organotypic cortex cultures in vitro [2], in rat cortical layer 2/3 in vivo under urethane anesthesia [4], in superficial cortical layers in an awake monkey with a low-density microelectrode array [5] and in another monkey with a high-density microelectrode array (Figure 1). The results will be presented in the following order: First, finite-size scaling analysis, which is required to determine whether the power law model is an appropriate model for neuronal avalanches. Second, parameter value estimation, which is an essential step in model selection and comparison as proper parameter estimates are required for any further quantitative analysis. We estimated the power law exponent for neuronal avalanches by two different methods, i.e., likelihood maximization and estimation based on the KS statistic, and we compared the results to previous reports that were obtained by least-square regression. Finally, we compared the power law model to alternative distributions by performing a log-likelihood ratio test [14], [18], [20] and, additionally, by using the KS statistic.

### Finite-size scaling in neuronal avalanches

An important feature of systems at a critical transition is the scale invariance of their dynamics with respect to changes of the system size [1], [6]. In neuronal avalanches, the distribution of cluster sizes with slope 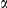 = −1.5 has been shown to be invariant to changes in the number of electrodes that was used for the avalanche detection [2], [5], [21]. This feature manifests non-trivial dynamics of the underlying network activity as the systematic removal of events in the local field potential (LFP) does not lead to a break-down of the avalanche size distribution (cf. [5]).

**Figure 2:**
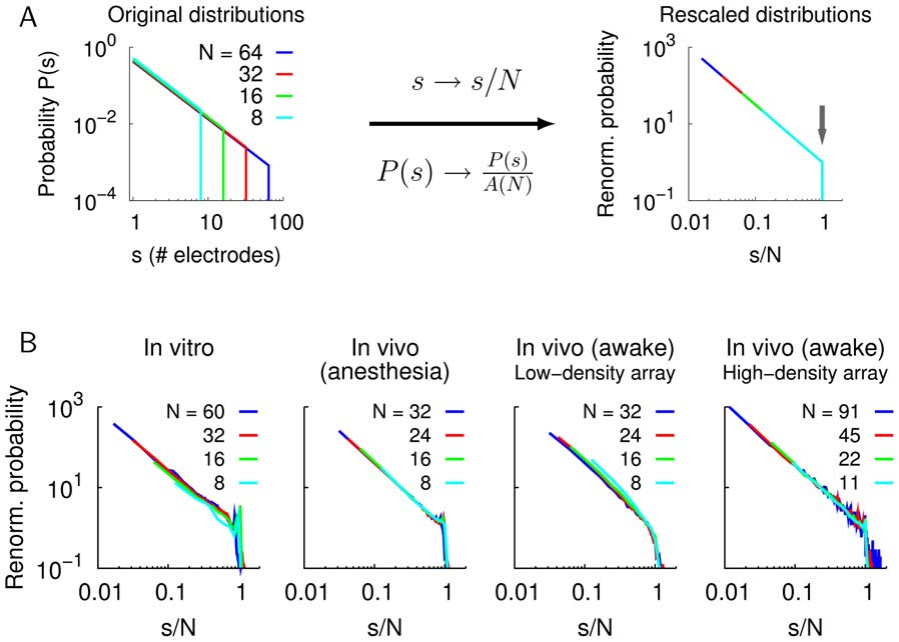
Collapse of rescaled cluster size distributions in neuronal avalanches. A. Depiction of the rescaling approach for synthetic PMFs for maximum sizes *N* = 8, 16, 32, 64 (left). The system size, *N*, corresponds to the number of electrodes included in the analysis. Cluster sizes *s* were normalized by the system size *N* (*s* 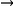 *s*/*N*) and the renormalized probability was obtained according to *P*(*s*)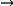 *P*(*s*)/*A*(*N*), resulting in a collapse of the cluster size distributions (right). Here, the definition of *A*(*N*) with upper bound *N* was used (Eq. 16). The vertical arrow indicates the system size (scaled to unity). B. Collapse of rescaled cluster size distributions for average in vitro distributions (*n* = 7), average in vivo distributions under anesthesia (rat, *n* = 7), and the two awake monkeys with low- and high-density array, respectively (from left to right). Note that the maximum cluster size for all data sets increases with *N* with the distribution showing a clear cut-off beyond the system size (*s*/*N* = 1). The exponent 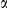 for the empirical distributions was fitted individually for each system size *N* (see *Materials and Methods*).

We used this property as an indicator of the power law scaling in neuronal avalanches. To study the invariance of cluster size distributions, we varied the number of electrodes, *N*, that were included for the detection of negative threshold crossings in the LFP (see *Materials and Methods*). Event sizes in the resulting size distributions were expressed in units of *N* by the basic rescaling approach *s* 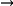 *s*/*N*. A proper renormalization of the probability mass functions (PMFs) resulted then in a collapse of power law distributions for different *N*, as shown in Figure 2A for theoretical power law distributions (see also Supporting Information, Text S1 and Figure S1). Figure 2B shows the collapse of cluster size distributions for the empirical data sets from Figure 1, indicating scale-free dynamics in neuronal avalanches independent of *N*. Importantly, the cluster size distributions for varying *N* showed a sharp cut-off at the system size (i.e., at *s*/*N* = 1) for the rescaled distributions (Figure 2B). Using a renormalization for time-shuffled cluster sizes based on either the power law assumption or the exponential model did not result in a collapse of the corresponding distributions (Figure S1). In addition, the maximum cluster size in the time-shuffled data decreased for increasing *N* (Figure S1), indicating that shuffling destroyed the scale-free behavior in the resulting distributions. These results, together with the finite-size scaling and the collapse of the original cluster size distributions in Figure 2 are consistent with critical state dynamics and the hypothesis of a power law distribution of neuronal avalanche sizes.

### Model parameter estimation

**Figure 3:**
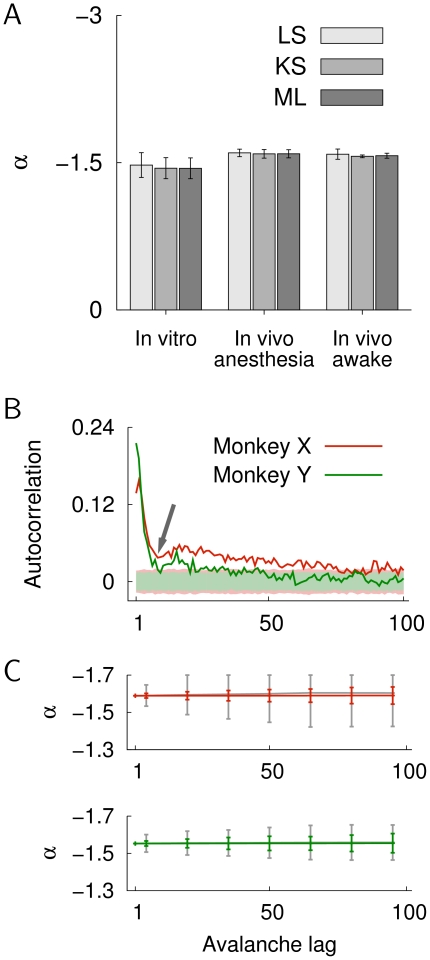
Estimation of slope parameter 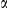 for the in vitro data (*n* = 7 cultures), the in vivo data under anesthesia (*n* = 7), and the in vivo recordings in awake monkeys (*n* = 2). A. Shown are the average slope parameters 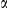 and the standard deviations (error bars). The three different estimation methods are: LS least-square estimation with logarithmic binning, KS Kolmogorov-Smirnov statistic, and ML maximum likelihood estimation (see *Materials and Methods*). Estimated values of 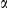 were not statistically different: in vitro, F(2,18) = 0.19, *p* = 0.827; in vivo (anesthesia), F(2,18) = 0.124, *p* = 0.884; in vivo (awake), F(2,3) = 0.21, *p* = 0.821 (one-way ANOVA). Values of 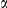 were estimated for the entire range of cluster sizes, i.e., from avalanches that included only one electrode to clusters that spanned the entire multielectrode array. B. Autocorrelation of the avalanche sizes for monkey X and Y as a function of the avalanche lag. The autocorrelation showed a fast decay within 10 avalanches (arrow). The shaded areas indicate the autocorrelation (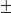
3 SD) for randomly permuted cluster sizes for both monkeys. C. Average 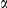 values obtained by ML estimation as a function of avalanche lag for monkey X and Y (red and green line, respectively). Error bars denote the standard deviation across the decorrelated sub-sets. The gray lines show mean
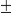
SD of ML parameter estimates for sample-size matched data from the original sequence of cluster sizes.

The slope parameter 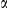 for neuronal avalanches has been previously reported to be close to −1.5. This value has been estimated by least-square (LS) fitting of a linear function on double-logarithmic plots with logarithmic binning [2], [5], an approach that has also been used for power law distributions in domains other than neuroscience (see, e.g., refs. [16], [17], [22]). If not handled with care, LS fitting on log-log transformed values can yield strongly biased estimates, originating from noise in the tail of the distribution or introduced by the bias of zero frequencies [11]–[13]. However, both issues did not pose a problem for the avalanche size distributions considered in the current study; the number of avalanches per experiment ranged from approximately 12,000 to >150,000, thus, providing numerous samples per size *s* up to the system size (Figure 1). With proper binning, this results in enough samples per bin even at the tail of the distribution. To assess the performance of the LS fit for the size distribution of neuronal avalanches, we calculated estimates of 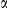 by applying two different methods, i.e., by minimizing the KS distance (Eq. 12) between the cumulative distribution of the empirical data set and the power law model, and, additionally, by using the maximum likelihood (ML) estimation (Eq. 15, refs. [14], [18], [20]). Due to the sharp cut-off in the cluster size distributions at the system size, *N*, which was the number of electrodes in the recording array (see Figure 1 and 2), parameter estimation was performed over the finite range of cluster sizes from *s* = 1 to *N* (total number of electrodes in the array). For all avalanche size distributions, average slope parameters 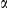 estimated by the three methods (LS, KS and ML) were close to −1.5 and not significantly different from each other (Figure 3A).

Since the ML estimation used here (Eqs. 13 to 15) assumes independently distributed cluster sizes, we verified that the temporal correlation structure in neuronal avalanches [21] did not have a significant influence on the parameter estimates of 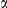. This was done by estimating 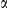 for decorrelated sub-sets of the data and comparing it to estimates that were obtained from sample-size matched sequences from the original cluster sizes. Figure 3B shows the autocorrelation for the sequence of neuronal avalanche sizes in monkey X and Y. Both correlation functions showed a quick drop within the first 10 avalanches (Figure 3B, arrow), followed by a slower, subsequent decay. Here, “decorrelation” refers to the strong reduction of the autocorrelation, which was achieved by considering a minimum lag between avalanches that eliminated the initial peak in the autocorrelation. Importantly, estimates of 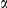 for the decorrelated avalanche sizes were not significantly different from estimates in sample-size matched controls (gray lines in Figure 3C) over a wide range of avalanche lags. Therefore, the ML estimation shown in Figure 3 gave reliable estimates for size distributions in neuronal avalanches, and ML estimation on decorrelated sub-sets of the data was used for the log-likelihood ratio test for the model comparison (see below). For all other tests, and if not stated otherwise, we used an estimation obtained by the KS method, which does not assume independently distributed data.

We also note that, when the empirical distribution was not well described by the model distribution, for example, when time-shuffled, exponentially distributed cluster sizes were fitted by a power law, a significant difference arose between the estimation methods (Figure S2). This implies that the similarity between the differently estimated power law exponents for neuronal avalanches was not due to a general property of the estimation methods. Rather, it suggests that the power law is an appropriate model for the data. Next, we compared the power law model to various alternative distributions by using the ML estimation in combination with a log-likelihood ratio test and the KS estimation for a comparison based on the KS statistic.

### Comparison of the power law model to alternative distributions

The collapse of cluster size distributions in Figure 2 suggests scale-free dynamics in neuronal avalanche sizes, which is a unique feature of the power law. Such a power law scaling indicates long-range correlations in the avalanche dynamics as opposed to random activity, which results in an exponential class distribution of cluster sizes. We therefore compared the power law with the exponential model for neuronal avalanches by a log-likelihood ratio (LLR) test (Eq. 17) using decorrelated data as described above. The LLR takes positive values if the likelihood of the power law model for a given empirical data set is larger than the likelihood of the exponential model, and it is negative if the likelihood of the exponential model is greater. The sign of the LLR can be used to determine which model should be favored if the LLR is significantly different from zero [14], [23]. We calculated the LLRs for decorrelated sub-sets of the data from Figure 1 and included the entire range of cluster sizes (i.e., from *s* = 1 to *s* = total number of electrodes in the array). All LLR values for the in vitro and the in vivo size distributions were positive and significantly greater than zero (range, 377–8269, *p*<0.0001), indicating that the power law was favored over the exponential distribution for all data sets tested (Table 1).

**Table 1:** Model comparisons using the LLR test for decorrelated sub-sets of the data.

	Power law vs. exponential	Truncated power law vs. lognormal	Truncated power law vs. gamma	Truncated power law vs. inv. Gaussian
**In vitro**	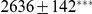	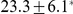	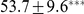	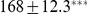
	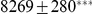	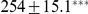	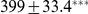	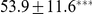
	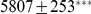	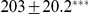	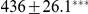	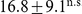
	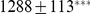	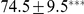	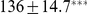	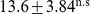
	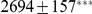	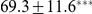	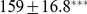	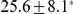
	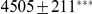	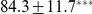	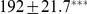	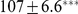
	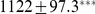	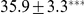	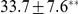	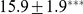
**In vivo (anesthesia)**	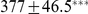	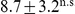	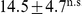	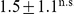
	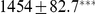	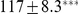	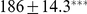	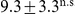
	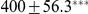	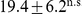	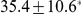	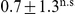
	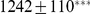	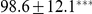	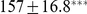	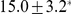
	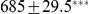	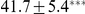	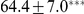	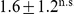
	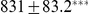	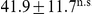	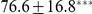	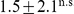
	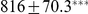	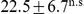	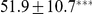	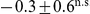
**In vivo (monkey X)**	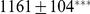	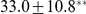	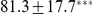	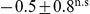
**In vivo (monkey Y)**	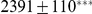	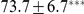	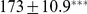	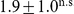

LLR test for the comparison of the power law with the exponential distribution, and of the exponentially truncated power law with the lognormal, the gamma and the inverse Gaussian distribution in all data sets (i.e., 7 data sets recorded in vitro, 7 in vivo under anesthesia, and 2 in vivo in awake monkeys). Data sets were decorrelated by choosing an avalanche lag for which the autocorrelation showed small values after the initial drop (cf. Figure 3B); the chosen avalanche lags were 4–26 (in vitro), 5–14 (in vivo, anesthesia), and 10 (monkey X and Y). LLR values for the model comparison in the decorrelated sub-sets are reported as mean
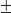
SD. Statistical significance (Eq. 18) is reported for the sub-set that had the largest *p*-value; 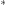 *p*<0.01, 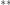 *p*<0.001, 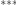 *p*<0.0001, n.s. not significant (*p* 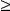
0.01).

**Figure 4:**
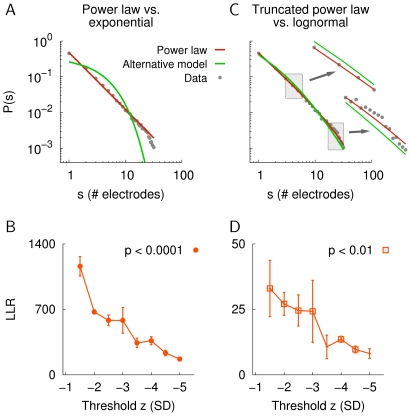
Model comparison using the LLR test. A. Model fits obtained by ML estimation for the power law (red) and the exponential model (green) for the cluster size distribution in monkey X. B. LLR values for increasing threshold, *z*. Error bars denote the SD across the decorrelated sub-sets of the data. All LLRs were positive and statistically different from zero (*p*<0.0001). The avalanche lags ranged from 10 (*z* = −1.5) to 2 (*z* = −5). C and D. The same for the comparison between the exponentially truncated power law (red) and the lognormal distribution as the alternative model (green). The insets show detailed views of the distributions, corresponding to the respective gray rectangles. The square symbols in (D) indicate the LLR values that were statistically different from zero (*p*<0.01).

It has been previously shown that the dynamics of neuronal avalanches exhibit scale-invariance with respect to changes of the threshold, *z*, of the nLFP detection [5]. Therefore, we compared the power law and the exponential model for decorrelated cluster sizes that were obtained for different nLFP thresholds in monkey X (cf. ref. [5]). The ML fits for the power law and the exponential distribution for *z* = −1.5 are shown in Figure 4A. Figure 4B shows the LLR values for *z* = −1.5 to −5 SD. All LLR values were positive and the difference from zero was highly significant (*p*<0.0001), indicating that the power law provided the better fit to the data. The drop of the LLR for more negative *z* was mainly due to the reduction of the number of avalanches per size distribution (see ref. [5]).

As evident from Figure 4A and Table 1, the exponential distribution cannot account for the distribution of neuronal avalanche sizes. The power law was therefore compared to the alternative of a lognormal distribution. Both the power law and the lognormal distribution are heavy-tailed, which can make them difficult to distinguish [15], [24]–[26]. In fact, the tail of the lognormal distribution can follow a linear relationship in double-logarithmic coordinates over a few decades when the dispersion parameter 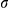 (Eq. 3) is large [15], [26]. Importantly, the single-parameter power law yielded significantly better fits for the majority of avalanche size distributions (Table S1). However, negative LLR values in those comparisons are difficult to interpret since the lognormal model has an additional degree of freedom (but see, e.g., ref. [23]). Therefore, to perform the LLR test between the power law and the lognormal distribution, we used the power law model with exponential cut-off (truncated power law), which also has two parameters (Eq. 4). The choice of the truncated power law is motivated by the fact that finite-size systems often show an exponential cut-off below the system size [6]. As evident from Table 1, the LLR values clearly favored the truncated power law over the lognormal model, i.e., all LLR values were positive and for the majority (12/16) of data sets this result was statistically significant. The model fits for the size distribution in monkey X are shown in Figure 4C (*z* = −1.5). Importantly, the distribution of cluster sizes according to a power law was maintained for more negative nLFP thresholds *z* (Figure 4D, monkey X).

In addition to the lognormal model, we tested two other heavy-tail distributions, i.e., the gamma and the inverse Gaussian distribution. The inverse Gaussian distribution describes a power law with fixed slope −1.5, and, compared to the truncated power law, it has a cut-off function with two parameters (Eq. 6). While the LLR test clearly favored the truncated power law over the gamma distribution for all cluster size distributions, the difference between the truncated power law and the inverse Gaussian distribution was statistically not significant for the majority of data sets (Table 1). For all size distributions tested here, we observed that the shape parameter, *k*, of the gamma distribution (Eq. 5) was close to zero, which means that the slope of the power law term in Eq. 5 was close to −1. Therefore, the model fits obtained for the gamma distribution were influenced by the constraint *k*>0 (Eq. 5), which explains the superiority of the truncated power law model. In contrast, the inverse Gaussian with fixed slope −1.5 provided fits that were comparable to the truncated power law (Table 1), indicating that neuronal avalanche size distributions were well described by a truncated power law with slope −1.5.

**Figure 5:**
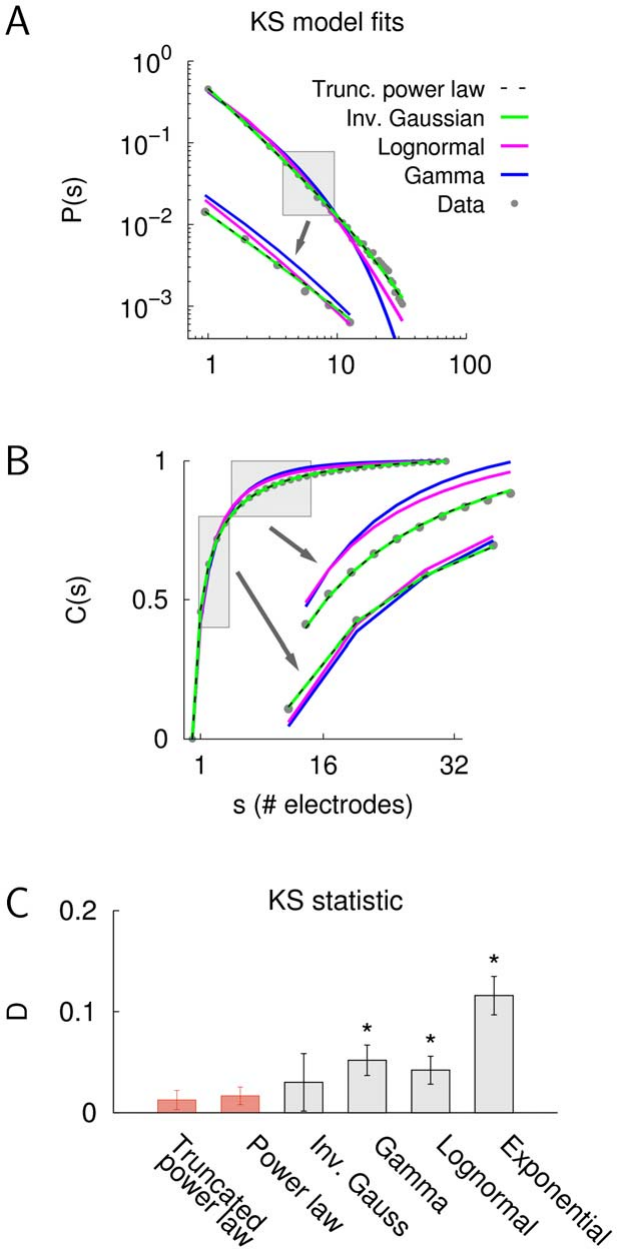
Model comparison based on the KS statistic. A. PMFs of the two-parameter models for the avalanche size distribution in monkey X. The inset shows a detailed view that corresponds to the gray rectangle. B. Corresponding CDF fits for the same size distribution (i.e., monkey X). The insets show detailed views of the distributions, corresponding to the respective gray rectangles. C. Average KS distance of the model distributions for all data sets (*n* = 16, which includes 7 data sets recorded in vitro, 7 in vivo under anesthesia, and 2 in vivo awake). Error bars denote the standard deviation. The single-parameter power law and the power law with exponential cut-off yielded significantly better fits to the data than the gamma, the lognormal, or the exponential distribution (Kruskal-Wallis test and Tukey-Kramer multiple comparison, *p*<0.0001).

In addition, we performed the comparison of the power law with and without exponential cut-off with the alternative distributions from Table 1 by using the KS statistic, *D* (Eq. 11). The KS fits for all two-parameter models for monkey X are shown in Figure 5A and B. The PMFs (Figure 5A) and the corresponding cumulative distribution functions (CDFs, Figure 5B) give a clear impression of the goodness-of-fit of the respective models, i.e., the truncated power law provided better fits to the data than the lognormal and the gamma distribution, whereas it was visually indistinguishable from the inverse Gaussian distribution. Furthermore, the average KS statistic, *D*, gave results that were in line with the LLR test (Table 1), that is, both the single-parameter and the truncated power law yielded significantly better fits compared to the gamma, the lognormal, and the exponential distribution (Figure 5C, *n* = 16, Kruskal-Wallis test and Tukey-Kramer multiple comparison, *p*<0.0001). In summary, these results indicate that the power law without and with exponential cut-off provide an excellent description of the size distribution in neuronal avalanches.

## Discussion

In the present study we showed that neuronal avalanches are consistent with the power law hypothesis of a scale-free distribution of avalanche sizes. We analyzed in vitro and in vivo recordings from various cortical areas, including somatosensory, primary motor and premotor cortex and for different species (rat and monkey). All cluster size distributions exhibited finite-size scaling, consistent with the scale-free property of a power law and with critical state dynamics [6]. Using both a maximum likelihood approach and minimization of the KS statistic to estimate the exponent of the power law, we found 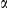 to be close to −1.5, which is in excellent agreement with earlier reports that were obtained by a linear regression on double-logarithmic coordinates. The power law scaling of neuronal avalanches was finally analyzed by a log-likelihood ratio test and a comparison based on the KS statistic, both of which favored the power law without and with exponential cut-off over the exponential, the lognormal and the gamma distribution.

### Parameter estimation

Simple parameter fitting approaches such as linear regression on logarithmic scales can lead to biased, and thus wrong, estimates [11], [12], [14]. Discrepancies between LS and ML estimates, which have been reported previously [12], [14], will be exacerbated by improper data binning. However, sufficient data together with proper data binning can provide relatively accurate parameter estimates by LS fitting. Accordingly, for neuronal avalanches, we found that average parameter values (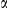 close to −1.5) were not significantly different between LS and ML estimation. It is important to note, however, that the bias in the LS estimation becomes evident when the assumed model is not consistent with the underlying empirical data, e.g., if time-shuffled, exponentially distributed data is fitted by a power law distribution (Figure S2).

Therefore, although we showed that the LS estimation can give accurate estimates, the KS and ML approach should be used when possible due to their more preferable properties [11]–[14].

### Identifying power law scaling in neuronal avalanches

The result of the parameter estimation yields values that specify the best fit of the proposed model to the empirical data, yet the estimated values do not give any information about the validity or the goodness-of-fit of the underlying model. Visual inspection and a subsequent model comparison are therefore essential steps during the process of model validation. However, a direct comparison of the hypothesized and an alternative model can be misleading if the alternative distribution only poorly fits the data. In such a case, a better fit of the data by the hypothesized distribution than by the alternative one does not mean that the hypothesized distribution is a good model [14]. Therefore, the hypothesized model has to be justified. Clauset et al. [14] proposed a solution to this conceptual problem and suggested a test that uses synthetic power law data to derive a distribution of KS values which is then compared to the KS statistic of the empirical distribution. A significant fraction, *p*, of synthetic data that has a larger deviation from the power law than the empirical distribution is interpreted as evidence that the empirical data set is consistent with the power law hypothesis (in ref. [14], the criterion was *p*>0.1). However, the failure to reject the null-hypothesis that an empirical distribution follows a power law, i.e., *p*>0.1 as suggested in ref. [14], does not necessarily mean that the null-hypothesis of a power law distribution is verified. It is possible that the sample size was not sufficiently large to detect possible deviations from a power law distribution (see Supporting Information, Text S2 and Figure S3). In fact, as any empirical, complex system does rarely follow an idealized, mathematical relation, it is reasonable to expect that with large enough sample size, the deviation from a power law – no matter how small and practically negligible – will eventually result in the rejection of the power law hypothesis for most empirical systems. Therefore, the result of such a “goodness-of-fit” test is less informative in the large sample size regime. We also note that, while the KS distance reflects the closeness between the empirical and the model distribution, i.e., the goodness-of-fit, the *p*-value from the test by Clauset et al. [14] does not. In other words, while a smaller KS distance indicates that the model yields a better fit to the data, a smaller *p*-value is not equivalent with a better fit by the model because the *p*-value depends on the sample size of the empirical distribution (Figure S3). Therefore, to determine whether the power law can be considered an adequate model for the avalanche size distributions, we analyzed the KS statistic (along with visual inspection) and used the finite-size scaling as an indicator of the scale-free dynamics in neuronal avalanches [6], [21]. Excluding electrodes for the avalanche detection results in a sharp cut-off at the system size (number of electrodes) but preserves the power law scaling in the resulting cluster size distributions [2], [5]. This scale-invariance was evident from the collapse of the renormalized cluster size distributions (Figure 2), and, together with theoretical predictions [2], [6], [8], it suggests the power law as a candidate model for these distributions. In line with this, the power law with and without exponential cut-off resulted in small values of the KS statistic for the cluster size distributions in neuronal avalanches.

### The power law model for neuronal avalanches is favored over the exponential and the lognormal and gamma distribution

Verifying the power law hypothesis for empirical data has been generally difficult and in some cases controversial [14], [18]. Some of the controversies were caused by methodological differences, e.g., by the use of a test that is dominated by the center of a distribution versus the tail [15], [24], [25], [27]. Other discrepancies might stem from the properties of the data sets; for example, the lack of power law scaling of activity bursts in cortical networks [19] can be a result of the recording depth and/or the limited number of electrodes (given possible finite-size effects in the system).

The comparison between two models becomes more challenging when the goal is to compare two distributions that share some characteristics. This is for example the case for the power law and the lognormal distribution, both of which are heavy-tailed [15], [26]. Under such circumstances sufficient data is required in order to reach statistically significant conclusions. For neuronal avalanches with tens of thousands of samples, the LLR values strongly supported the exponentially truncated power law when compared to the lognormal and the gamma distribution. In addition, despite the fact that the single-parameter power law had only one degree of freedom, it provided better fits to most cluster size distributions than the lognormal or the gamma distribution (Table S1 and Figure 5C).

In the present study, we confirmed the power law scaling in neuronal avalanches with slope parameter −1.5 for previously published data sets and in a new high-density recording in an awake monkey. The finite-size scaling together with the results from the model comparison provide further support for critical state dynamics in cortical networks. That neuronal avalanches reflect critical dynamics in cortical networks is further supported by the critical branching parameter that captures the evolution of an avalanche [2] as well as the optimal properties networks with neuronal avalanches attain [10], [28] that are in line with theoretical predictions from criticality [29]. Importantly, neuronal avalanches reflect critical dynamics actively regulated by cortical networks that break down when network parameters are changed, for example, by acute pharmacological manipulations of synaptic transmission [2]–[4], [10]. In this context, the question is to a lesser extent whether or not a given distribution follows exactly a power law, but how strong the deviation from a power law is. In fact, recent experimental evidence allows the quantitative deviation of ongoing activity from neuronal avalanche dynamics to be used as a predictor of its dynamic range [10] and information capacity [28]. We suggest that the identification of power law scaling in neuronal avalanches as shown in the present study and the proper quantification of the deviation from the power law [10], [28] might be a useful diagnostic tool for normal and abnormal cortical dynamics.

## Materials and Methods

### Multielectrode recordings

All animal procedures were in accordance with National Institutes of Health guidelines. Animal procedures were approved by the Animal Care and Use Committee at Duke University for monkey X (see below) and by the National Institute of Mental Health Animal Care and Use Committee for all other data sets.

The avalanche size distributions for the in vitro data was taken from [2]. In short, organotypic cortex slice cultures were grown on 60-channel planar multielectrode arrays (interelectrode distance, 200 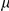
m; electrode diameter, 30 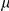
m; Multichannelsystems, Reutlingen, Germany). Extracellular signals were sampled at 1 kHz and the local field potential (LFP) was low-pass filtered at 50 Hz. Negative LFP (nLFP) peaks were extracted by applying a threshold, *z*, that was calculated based on the standard deviation (SD) of the LFP signals (*z* = −3 SD).

Spontaneous in vivo data under urethane-anesthesia was taken from [4]. Neuronal activity was measured in rat cortical layer 2/3 at the end of the second week postnatal (P13
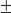
2, *n* = 7). An 8
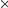
4 microelectrode array (interelectrode distance, 200 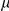
m; Neuronexus Technologies, Ann Arbor, MI, USA) was inserted 1 mm deep into the somatosensory cortex to record spontaneous LFP activity (1–200 Hz band pass filter; 4 kHz sampling frequency; threshold for nLFP detection, *z* = −3 SD).

For the avalanche size distribution in awake monkey (monkey X), we used the data published in [5]. In short, 43 min of ongoing LFP activity (band-pass filtered at 1–100 Hz) was recorded from a customized 64-channel array implanted in the left motor cortex of an adult, male rhesus monkey (*Macaca mulatta*). The array consisted of tungsten electrodes with 30 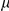
m diameter, 1 M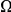 impedance, and 1-mm spacing between the electrodes. LFP activity was recorded from every other electrode. For the precise layout of the array see [5]. If not stated otherwise, the threshold for nLFP detection was *z* = −1.5 SD.

The data for monkey Y was recorded by a 96-channel high-density microelectrode array (91 working channels; interelectrode distance, 400 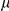
m; electrode length, 1 mm; electrode impedance, 200–600 k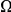; Blackrock Microsystems, Salt Lake City, UT, USA) that was chronically implanted in the left premotor cortex (*Macaca mulatta*, adult female). For a detailed description of the surgical and behavioral procedure see Supporting Information (Text S3) and ref. [28]. Ongoing activity was recorded for 30 min while the animal was sitting in a primate chair, alert but not engaged in any behavioral task. LFP signals were band-pass filtered at 1–100 Hz (nLFPs threshold, *z* = −2.5 SD).

### Detection of neuronal avalanches

Rasters of nLFP events that crossed a predefined threshold, *z*, were created by binning the nLFP times with bin size 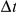. From the nLFP rasters, neuronal avalanches were extracted by finding clusters of nLFP events that were separated by at least one bin width. The size of a neuronal avalanche was defined as the number of active electrodes during a cluster. Multiple electrode activations were counted if an electrode was activated more than once during a cluster. Therefore, the size of a neuronal avalanche is equivalent to the number of nLFPs during the avalanche. The threshold for the nLFP detection, *z*, and the bin size, 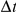, were: −3 SD and the average event interval for individual recordings (in vitro and in vivo under anesthesia, refs. [2] and [4], respectively), −1.5 SD and 4 ms (monkey X, ref. [5]), and −2.5 SD and 2 ms (monkey Y), respectively. The neuronal avalanche size distributions are invariant with respect to *z* (see ref. [5] and Figure 4). For the choice of 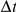, see refs. [2], [4], [5].

### Time-shuffled data

Time-shuffled versions of nLFP rasters were obtained by random permutation of bins for individual channels, while keeping the total number of nLFPs per electrode unchanged (i.e., rate-matched schuffling).

### Model distributions

For the cluster size distributions in neuronal avalanches, we tested the power law model – indicative of long-range spatiotemporal correlations – against the alternative of an exponential distribution, which would be expected from uncorrelated, random activity (for an identical rate between channels it would be the binomial distribution). We also compared the power law to the lognormal distribution as both are heavy-tailed, a property that can make them difficult to distinguish [15], [24]–[26]. In addition, we performed a comparison for the power law with exponential cut-off (“truncated” power law), the gamma and the inverse Gaussian distribution.

#### Power law distribution

The probability mass function (PMF) for the discrete power law (Pareto distribution) is
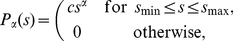
(1)with exponent, i.e., slope parameter, 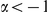. For the probability functions, we use the parameter symbols as index to denote the corresponding model, which for the power law is the symbol 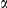. The constant 
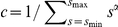
 normalizes the PMF, such that 
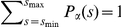
. The definition of the theoretical PMF in Eq. 1 requires a lower bound 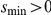, since 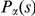 diverges for 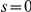, but can be written with an upper bound 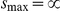. For empirical data, however, an upper bound is always given by the largest sample in the distribution. The expected size of the largest sample for a scale-free distribution depends on the exponent 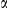 and the number of samples [13], [18]. Adjusting 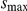 to the largest sample in the distribution is required for a proper normalization of the PMF [18]. However, for the neuronal avalanche size distributions in this study, 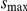 will be determined by the finite system size, i.e., the finite number of electrodes in the recording array (in the cases considered here, 27 to 91 channels).

For many real systems, the data will not follow the hypothetical distribution – such as a power law – over the entire range of sampled values [6], [13], [14]. Therefore, only a given range of sizes 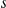 can be considered, that is, for 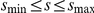. For a discussion on how to determine the lower bound, see [14]. In this study, parameter estimates and log-likelihood ratios (see below) are reported for the range of sizes from 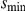 = 1 to 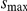 = total number of electrodes in the array. In any case, the probability function in the range 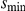 to 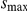 has to be normalized to unity for both the empirical and theoretical PMF.

#### Exponential distribution

The PMF for the exponential distribution with parameter 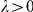 is
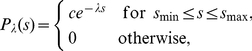
(2)with normalization constant 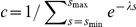.

#### Lognormal distribution

The PMF of the lognormal distribution is given by
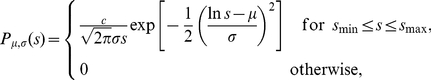
(3)with dispersion parameter 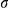, location parameter 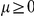 and proper normalization 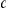.

#### Power law distribution with exponential cut-off

The power law distribution with exponential cut-off (“truncated” power law) is given by
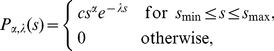
(4)with 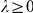 and proper normalization constant 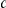.

#### Gamma distribution

The PMF of the gamma distribution is given by
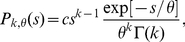
(5)with shape parameter 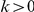, scale parameter 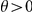, and proper normalization constant 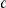. The gamma function is defined as 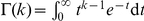.

#### Inverse Gaussian distribution

The PMF of the inverse Gaussian distribution is given by
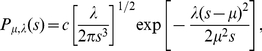
(6)with mean 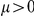, shape parameter 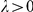, and proper normalization constant 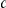. Note that the inverse Gaussian distribution is essentially a power law (slope exponent −1.5) with a cut-off that is given by the exponential term in Eq. 6. Therefore, the comparison between the truncated power law (Eq. 4) and the inverse Gaussian distribution (Eq. 6) is not a test whether or not a distribution follows a power law, but mainly which of the exponential cut-off terms yields a better fit to the data. The difference between the cut-off terms in both models is that the cut-off in Eq. 4 is monotonically decreasing, whereas this is not generally the case for the inverse Gaussian distribution.

#### Empirical distribution

The empirical PMF for an observation, 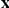, with 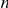 discrete samples, 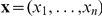, is given by
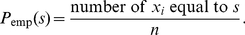
(7)


#### Cumulative distribution

The Kolmogorov-Smirnov estimation (see below) is based on the cumulative distribution function (CDF) rather than the PMF. For a given probability density, 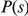, the corresponding CDF is
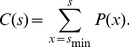
(8)


### Parameter estimation

#### Least square (LS) fit

For a vector of discrete cluster sizes, 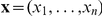, data was logarithmically binned between 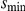 and 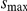 (10 bins), i.e., the binned probability function, 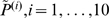, was normalized by the width of the *i*-th bin. The logarithms of 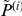 were then used for a linear least-square fit. The resulting objective function for the power law model with slope parameter 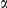 is
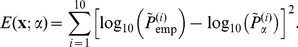
(9)


We write the objective function 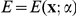 as a function of the data 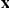 since the PMF, which is used in Eq. 9 depends on 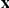. However, to calculate 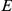 for parameter 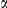, it is sufficient if only the PMF and not the original vector of cluster sizes is available. An estimate for 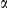 that best fits data 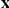 was obtained by minimizing 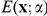 in Eq. 9:
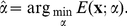
(10)


Parameter estimates for the other models were obtained analogously (Eqs. 9 and 10).

#### Kolmogorov-Smirnov (KS) estimation

The KS-statistic is based on cumulative distribution functions. For the empirical CDF of data 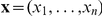, 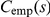, and a power law distribution, 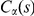, the KS-statistic is defined as
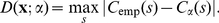
(11)


Again, minimizing the objective function in Eq. 11 yields an estimate for the slope parameter 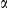 of the power law model (estimates for other model distributions can be obtained analogously):
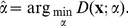
(12)


#### Maximum likelihood (ML) estimation

The likelihood of the power law model with parameter 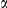, given the sample of cluster sizes, 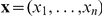, is
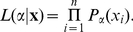
(13)


For numerical convenience, maximum likelihood and likelihood ratios are calculated with logarithmically transformed values of 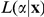. The log-likelihood is given by
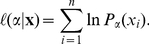
(14)


An estimate, 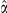, of the power law exponent for data 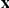 can then be obtained by maximizing the log-likelihood function in Eq. 14 (see, e.g., refs. [30] and [31]):
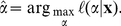
(15)


Maximum likelihood estimates for all other models can be obtained analogously (Eqs. 13 to 15).

If not stated otherwise, parameter values were estimated for the entire range of cluster sizes, i.e., from avalanches that included only one electrode to clusters that spanned the entire multielectrode array (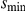 = 1, 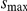 = total number of electrodes in the array).

For the minimization of Eqs. 10 and 12 and the maximization of Eq. 15, we applied the Nelder-Mead method [32]. Here, the *fminsearch* implementation in Matlab was used. For all models, different initial values were tested and the algorithm was tested for convergence. For the power law, e.g., initial conditions between −1 and −2 were found to give the same optimal solution. To assure the validity of the optimization results, objective functions were also studied by a grid search method for a wide range of parameter values.

### Finite-size scaling analysis

In scale-free systems, the maximum event size is not limited by the dynamics of the system but only by the system's finite size [1], [6]. We systematically varied the number of channels for the avalanche detection and studied the probability distribution of normalized cluster sizes, 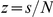, where 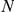 denotes the finite number of channels in the (sub-) array. Rescaled sizes 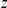 are expressed in units of system size 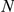 and are no longer integers.

Here, we write the PMF for 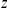 as 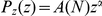, where 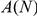 is the normalization factor that depends on 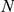. With 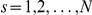 and the property
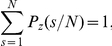
one obtains
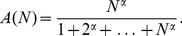
(16)


Dividing 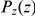 by 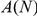 gives 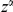, which is independent of 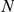. Thus, the transformation 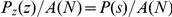 results in a collapse for power law distributions with slope parameter 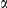, where 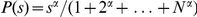 denotes the normalized PMF for cluster sizes 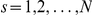. For a derivation of this result and of Eq. 16, see the Supporting Information (Text S1). For the empirical distributions, we fitted the slope parameter 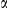 in Eq. 16 individually for each system size 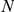 (KS estimation).

### Log-likelihood ratio test

The log-likelihood ratio for the power law and exponential distribution was defined as
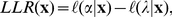
(17)where 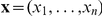 is the sample of cluster sizes, and the 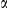 and 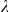 are ML estimates of the power law and exponential distribution, respectively (cf. Eq. 15). If 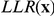 is significantly larger than zero then the power law is considered to be the better model for data 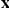 when compared to the exponential distribution. Conversely, if 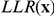 is significantly smaller than zero, the exponential distribution is the better fit. The *p*-value for the LLR test is given by
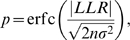
(18)where
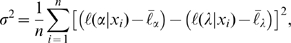
with 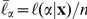 and 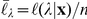 [14]. Here, we used a significance level of 0.01. The LLR for the comparison of the truncated power law with the other model distributions can be calculated analogously (Eqs. 17 and 18).

### Autocorrelation function

The autocorrelation, 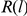, of the sequence of avalanche sizes as a function of the avalanche lag, 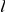, was measured as follows:
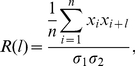
(19)where 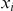, 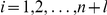, denotes the mean-subtracted avalanche sizes, and 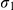 and 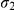 the standard deviations of the sequences 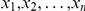 and 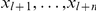, respectively. 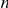 is the number of avalanches in the entire sequence minus the lag 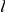.

## Supporting Information

Text S1 Finite-size scaling analysis.
(PDF)

Text S2 Goodness-of-fit evaluation as suggested by Clauset et al. [14].
(PDF)

Text S3 Surgical and behavioral procedure for monkey Y.
(PDF)

Figure S1Rescaled cluster size distributions for time-shuffled data do not collapse. A. Unscaled PMFs of time-shuffled cluster sizes for different system sizes in the high-density array of monkey Y (*N* = 11, 22, 45, 91). B. Renormalized PMFs assuming a power law distribution, i.e., *P*(*s*)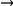 *P*(*s*)/*A*(*N*) with 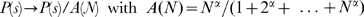 (Eq. 16). C. Renormalized PMFs assuming the exponential distribution with 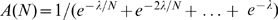 (see Supporting Information, Text S1). Cluster sizes in B and C were normalized by the system size *N* (indicated by the gray arrows at unity).(TIF)

Figure S2Parameter estimation for the original and the time-shuffled data in two data sets (monkey X and Y). A. Power law model with slope parameter 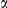. B. Exponential model with parameter 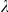. Three different estimation methods were compared: LS least-square estimation, KS Kolmogorov-Smirnov statistic, and ML maximum likelihood estimation. Note that all estimation methods yield similar estimates for the power law fit of the original distributions and the exponential fit of the time-shuffled distributions. However, LS estimation gave largely different values compared with KS and ML estimation when the original distribution was fitted by an exponential model, or when the power law was assumed for the time-shuffled data. The error bars denote the standard deviation for parameter estimates that were obtained by bootstrapping (200 synthetic data sets were drawn from the empirical distribution and their corresponding parameters were estimated). In some cases, the error bar is too small to distinguish.(TIF)

Figure S3Sample size dependency of the Clauset et al. [14] goodness-of-fit evaluation. A and B. Avalanche size distribution in monkey X for a sub-set with *n* = 1000 and for the whole data set with *n* = 45,548 avalanches, respectively. Shown are the empirical PMFs, 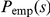 (gray), and the best-fit power law distributions, 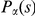 (red). C. Average *p*-value for different sample sizes in the empirical data (gray line). The gray area indicates the *p*-values between the 5th and 95th percentile. The *p*-value for a synthetic power law is uniformly distributed on the interval (0,1) with an average value close to 0.5 (blue line). D–F. The same for the time-shuffled data set and the exponential distribution as the model distribution (green). Note that both empirical distributions (i.e., original and rate-matched, time-shuffled data) will eventually fail against synthetic data sets given the perfect convergence of the synthetic distributions towards the model distribution for increasing *n*.(TIF)

Table S1 Comparison of the single-parameter power law with the lognormal and the gamma distribution using the LLR test for decorrelated data.
(PDF)
