# Supplementary material for: Statistical Analyses Support Power Law Distributions Found in Neuronal Avalanches
Source: PLoS One. 2011 May 26;6(5):e19779. doi: 10.1371/journal.pone.0019779 (PMC3102672; doi:10.1371/journal.pone.0019779)
Supplement: Text S1 — Finite-size scaling analysis. (PDF) [file pone.0019779.s001.pdf]

Derivation of A(N) in Eq. 16 (from the main text)

The (normalized) probability mass function (PMF) for a power law distribution of cluster sizes $s = 1, 2, \dots, N$ is

$$P(s) = \frac{s^\alpha}{1 + 2^\alpha + \cdots + N^\alpha}. \quad (1)$$

For the PMF of rescaled sizes, $z = s/N$, we write

$$P_z(z) = A(N) z^\alpha, \quad (2)$$

where $A(N)$ denotes the normalization factor that depends on the sytem size N. From Eq. 2 and with the property

$$\sum_{s=1}^{N} P_z(s/N) = 1,$$

one obtains

$$A(N) \left[\left(\frac{1}{N}\right)^\alpha + \left(\frac{2}{N}\right)^\alpha + \cdots + \left(\frac{N}{N}\right)^\alpha \right] = 1,$$

and thus

$$A(N) = \frac{N^\alpha}{1 + 2^\alpha + \cdots + N^\alpha}, \quad (3)$$

which is equivalent to Eq. 16 from the main text.

Analogously, for the exponential distribution $P_z(z) = A(N) e^{-\lambda z}$ with $z = s/N$ ($s = 1, \dots, N$), one obtains

$$A(N) = \frac{1}{e^{-\lambda/N} + e^{-2\lambda/N} + \cdots + e^{-N\lambda/N}}. \quad (4)$$

For the interested reader, we also provide the results for the continuous power law distribution. The probability density function for cluster sizes $b \leq s \leq N$ and exponent $\alpha < -1$ is given by

$$P(s) = \frac{\alpha + 1}{N^{\alpha+1} - b^{\alpha+1}} s^\alpha, \quad (5)$$

where b denotes the lower bound for s. The normalized probability density for a rescaled system of size N is defined as

$$P_z(z) = A(N) z^\alpha. \quad (6)$$

Since s ranges from b to N, the rescaled size z ranges from b/N to 1. This gives the following constraint for $P_z(z)$:

$$\int_{b/N}^{1} P_z(z) \mathrm{d}z = 1.$$

From this and with $\alpha < -1$, it follows that

$$\int_{b/N}^{1} A(N) z^\alpha \mathrm{d}z = \left[\frac{A(N)}{\alpha + 1} z^{\alpha+1} \right]_{b/N}^{1} = \frac{A(N)}{\alpha + 1} \left[1 - (b/N)^{\alpha+1} \right] = 1,$$

which yields

$$A(N) = \frac{\alpha + 1}{1 - (b/N)^{\alpha+1}}. \quad (7)$$

Collapse of cluster size distributions

Here, we show that the transformation $z = s/N$ and a proper rescaling of $P(s)$, $s = 1, 2, \ldots, N$, results in a collapse of power law distributions for different system sizes N. From $z = s/N$ and Eqs. 1 to 3 it follows that $P_z(z) = P(s)$. Dividing $P_z(z)$ (Eq. 2) by $A(N)$ (Eq. 3) gives z^α, which is independent of N. The rescaling for the discrete power law distribution is therefore given by $z = s/N$ and $P_z(z)/A(N) = P(s)/A(N)$. Consequently, the rescaled probability

$$\frac{P(s)}{A(N)} = \frac{s^\alpha}{1 + 2^\alpha + \cdots + N^\alpha} \frac{1 + 2^\alpha + \cdots + N^\alpha}{N^\alpha} = \frac{s^\alpha}{N^\alpha}$$

is the same for a rescaled system with $z = s/N = (ks)/(kN)$, that is, for system size kN and cluster size ks:

$$\frac{P(ks)}{A(kN)} = \frac{(ks)^\alpha}{1 + 2^\alpha + \cdots + (kN)^\alpha} \frac{1 + 2^\alpha + \cdots + (kN)^\alpha}{(kN)^\alpha} = \frac{(ks)^\alpha}{(kN)^\alpha} = \frac{s^\alpha}{N^\alpha}.$$

Therefore, a power law with exponent α results in a collapse of $P(s)/A(N)$ for different N.

For the continuous power law distribution, the change in variables, $z = s/N$, yields $P_z(z) = P(s)N$. The proper rescaling of the probability density is therefore given by $z = s/N$ and $P_z(z)/A(N) = P(s)N/A(N)$. Rescaling of the probability density $P(s) = (\alpha + 1)/(N^{\alpha+1} - b^{\alpha+1})s^\alpha$, $b \le s \le N$ (Eq. 5) for system size N and cluster size s yields

$$\frac{P(s)N}{A(N)} = \frac{\alpha + 1}{N^{\alpha+1} - b^{\alpha+1}} s^\alpha N \frac{(N^{\alpha+1} - b^{\alpha+1})/N^{\alpha+1}}{\alpha + 1} = \frac{s^\alpha}{N^\alpha},$$

which is equal to the value one obtains for a rescaled system with $z = (ks)/(kN)$, that is, for system size kN and cluster size ks:

$$\frac{P(ks)kN}{A(kN)} = \frac{\alpha + 1}{(kN)^{\alpha+1} - b^{\alpha+1}} (ks)^\alpha kN \frac{((kN)^{\alpha+1} - b^{\alpha+1})/(kN)^{\alpha+1}}{\alpha + 1} = \frac{s^\alpha}{N^\alpha}.$$

Therefore, a continuous power law with exponent α results in a collapse of $P(s)N/A(N)$ for different N (the same result can be obtained for a power law distribution with an upper bound ∞).
