# Supplementary material for: Statistical Analyses Support Power Law Distributions Found in Neuronal Avalanches
Source: PLoS One. 2011 May 26;6(5):e19779. doi: 10.1371/journal.pone.0019779 (PMC3102672; doi:10.1371/journal.pone.0019779)
Supplement: Text S2 — Goodness-of-fit evaluation as suggested by Clauset et al. [14]. (PDF) [file pone.0019779.s002.pdf]

Goodness-of-fit evaluation

Here, we calculated the p-value for the goodness of fit as proposed by Clauset et al. [1] with the difference that we consider an upper bound $s_{\max}$ (see *Materials and Methods*), which is necessary for the correct normalization of the distribution functions as sampled data was obtained from finite-size systems [2]. Here, $s_{\max}$ was the number of channels of the multielectrode array. The p-value for the goodness-of-fit evaluation is defined as the fraction of synthetic sample distributions with a KS distance to the best-fit power law that is larger than the KS distance between the empirical distribution and its best-fit power law model [1]. A smaller p-value is interpreted as a poorer fit by a power law distribution. For distributions that strongly deviate from a theoretical power law, the p-value will approach zero. However, for synthetic power law data, p will be distributed on the interval $(0, 1)$ with the average converging to 0.5 as the number of synthetic data sets increases [1]. Thus, using for example $p \leq 0.05$ as a criterion for the rejection of the power law hypothesis means that 5% of (synthetic) power law distributions would be rejected as such (which corresponds to the type I error given the power law as the null-hypothesis). Conversely, if the p-value is larger than the given threshold, the power law hypothesis cannot be rejected.

To get sufficiently accurate p-values, enough synthetic distributions have to be calculated for the above comparison. Here, we used 2,000 synthetic distributions, which gives accurate values to the second decimal place [1]. For each synthetic distribution, best-fit model parameters were individually estimated.

The p-value test compares an empirical data set to synthetic distributions that only suffer from finite sample noise (under the assumption of independent and identically distributed data). Such sampling noise can be simply reduced by increasing the sample size. Any additional source of variability – no matter how small – could therefore be detected by a sufficiently large number of samples. For small sample sizes, however, existing deviations from a power law might not be detectable. In their analysis of 24 empirical data sets, Clauset et al. [1] reported that 17 distributions passed the goodness-of-fit evaluation with $p > 0.1$. Importantly, 9 out of 17 distributions that passed the goodness-of-fit test in ref. [1], had fewer than 500 samples in the tail of the distribution that was used for the analysis, and only one data set out of 17 had more than 3500 samples. In contrast, a neuronal avalanche size distribution from a 30−60 min recording typically contains tens of thousands of avalanches (Figure 1, main text), which, together with the finite range of sizes, gives a large number of samples per cluster size s. Figure S3A and B show the probability distribution in monkey X for a sub-set of 1000 avalanches — with the tail of the distribution strongly affected by finite sampling fluctuations — and the entire data set with >45,000 avalanches, respectively. For sample sizes $n \sim 100$, the average p-value is larger than 0.4 (Figure S3C, gray line). For $n \sim 500$, the majority of empirical distributions still has a p-value larger than 0.1. However, the p-value decreases rapidly as the sample size n further increases (Figure S3C). Importantly, a similar behavior was observed for time-shuffled, rate-matched control data and the exponential model distribution (Figure S3D−F). In comparison to the empirical distributions, the average p-value for synthetic data is around 0.5 independent of sample size (Figure S3C and F, blue lines). Therefore, any empirical data that suffers from more than finite sampling noise will eventually fail the p-value test for increasing n when compared to synthetic distributions.

Generating synthetic distributions

All random numbers were drawn from a uniform distribution on the interval (0,1) using Matlab's *rand* function. Random numbers for power law and exponentially distributed samples can be derived by applying the inverse method (see, e.g., ref. [3]). For the discrete distributions used in this study, we used a more efficient way of directly deriving synthetic PMFs. Given a cumulative distribution, $C(s)$, a synthetic probability distribution, $P_{\text{synth}}(s)$, that corresponds to a sample size of n (i.e., number of avalanches) can be calculated as follows: (i) draw a vector, $\mathbf{u}$, of n uniformly distributed random numbers on the interval

(0,1), (ii) calculate the probability of event size s as $P_{\mathrm{synth}}(s) = q_s - q_{s-1}$, where q_s denotes the fraction of samples in $\mathbf{u}$ smaller than $C(s)$ (for $s = s_{\min}, \ldots, s_{\max}$ with $q_{s_{\min}-1} \equiv 0$).

References

1. Clauset A, Shalizi CR, Newman MEJ (2009) Power-law distributions in empirical data. SIAM Review 51: 661–703.
2. Edwards AM, Phillips RA, Watkins NW, Freeman MP, Murphy EJ, et al. (2007) Revisiting Lévy flight search patterns of wandering albatrosses, bumblebees and deer. Nature 449: 1044–1048.
3. Newman MEJ (2005) Power laws, pareto distributions and Zipf's law. Contemporary Physics 46: 323–351.
