# Supplementary material for: Statistical Analyses Support Power Law Distributions Found in Neuronal Avalanches
Source: PLoS One. 2011 May 26;6(5):e19779. doi: 10.1371/journal.pone.0019779 (PMC3102672; doi:10.1371/journal.pone.0019779)
Supplement: Text S3 — Surgical and behavioral procedure for monkey Y. (PDF) [file pone.0019779.s003.pdf]

Surgical and behavioral procedure for monkey Y

Monkey Y was an adult female monkey (*Macaca mulatta*). All procedures were in accordance with NIH guidelines and were approved by the National Institute of Mental Health Animal Care and Use Committee. The monkey received an operation to place a titanium head restraint post by using standard sterile techniques. The operation was preceded by sedation with ketamine hydrochloride (10–20 mg/kg; intramuscular, i.m.), atropine injection (0.04 mg/kg, i.m.) to reduce oral secretions, and intravenous (i.v.) injection of antibiotics (30 mg/kg cefazolin). After hair removal and cleaning, a saline i.v. drip was established. Then, a surgical level of anesthesia was maintained with a gas inhalant (isoflurane, 1–4%). Heart rate (EKG), pCO_2, SPO_2 and body temperature were constantly monitored to judge the animal's anesthetic state. After the animal's head was fixed in a standard stereotaxic frame, the skin was incised. The skin, fascia and muscles were retracted to expose the skull. The titanium head restraint post (pre-sterilized) was attached to the skull by using 14 stainless steel screws. Then the fascia and skin were re-approximated around the restraint post. Antibiotic therapy was continued for 5 days after the operation. After this operation, the animal was given one week for recovery. Then, with the physical condition approved by a veterinarian, the animal was trained to stay in a primate restraining chair with the head restrained.

The second surgery for chronic multielectrode array implantation took place 4 weeks after the first surgery. The general procedure for the surgery was the same as described above. A 96-channel microelectrode array (10×10 configuration, 0.4-mm separation and 1.0-mm electrode length; from Blackrock Microsystems, Salt Lake City, UT, USA) was chronically implanted at the arm representation region of the left premotor cortex by using methods that have been described previously [1]. After the skull was exposed, a percutaneous connector was attached to the skull by using stainless steel screws. Craniotomy was performed over the premotor cortex of the left hemisphere. The dura mater was incised to expose the cortex. The silicon based array was then inserted into the target area by using a pneumatic impulse inserter (Blackrock Microsystems, Salt Lake City, UT, USA). The electrode array and cables connecting the electrodes to the percutaneous connector were sterilized prior to surgery. The reference wires were inserted in the subdural space. After that, the dura was re-approximated and sutured in place. Then the bone flap, which was stored in sterilized saline after the craniotomy, was replaced and sutured in place. The fascia and skin were re-approximated around the percutaneous connector in two layers. Antibiotic therapy was continued for 7 days after the operation.

The recordings took place one week after the surgery to allow the animal to recover. During recording, the monkey was sitting in a primate chair with head fixed. Ongoing activity was recorded for a period of 30 min, during which the monkey was awake but did not perform any behavioral task.

Reference

1. Suner S, Fellows MR, Vargas-Irwin C, Nakata GK, Donoghue JP (2005) Reliability of signals from a chronically implanted, silicon-based electrode array in non-human primate primary motor cortex. IEEE Transactions in Neural Systems and Rehabilitation Engineering 13: 524–541.
