# Supplementary figures and images for: Statistical Analyses Support Power Law Distributions Found in Neuronal Avalanches
Source: PLoS One. 2011 May 26;6(5):e19779. doi: 10.1371/journal.pone.0019779 (PMC3102672; doi:10.1371/journal.pone.0019779)

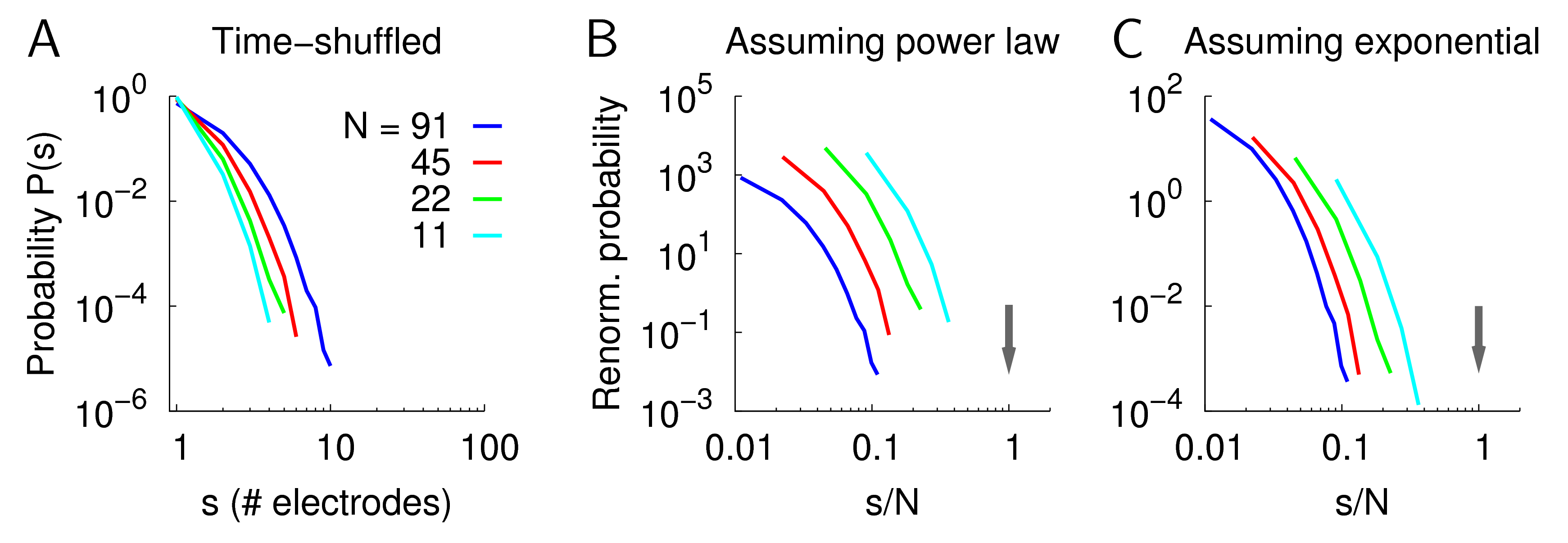

Supplement: Figure S1 — Rescaled cluster size distributions for time-shuffled data do not collapse. A. Unscaled PMFs of time-shuffled cluster sizes for different system sizes in the high-density array of monkey Y (N = 11, 22, 45, 91). B. Renormalized PMFs assuming a power law distribution, i.e., P(s) P(s)/A(N) with (Eq. 16). C. Renormalized PMFs assuming the exponential distribution with (see Supporting Information, Text S1). Cluster sizes in B and C were normalized by the system size N (indicated by the gray arrows at unity). (TIF) [file pone.0019779.s004.tif]

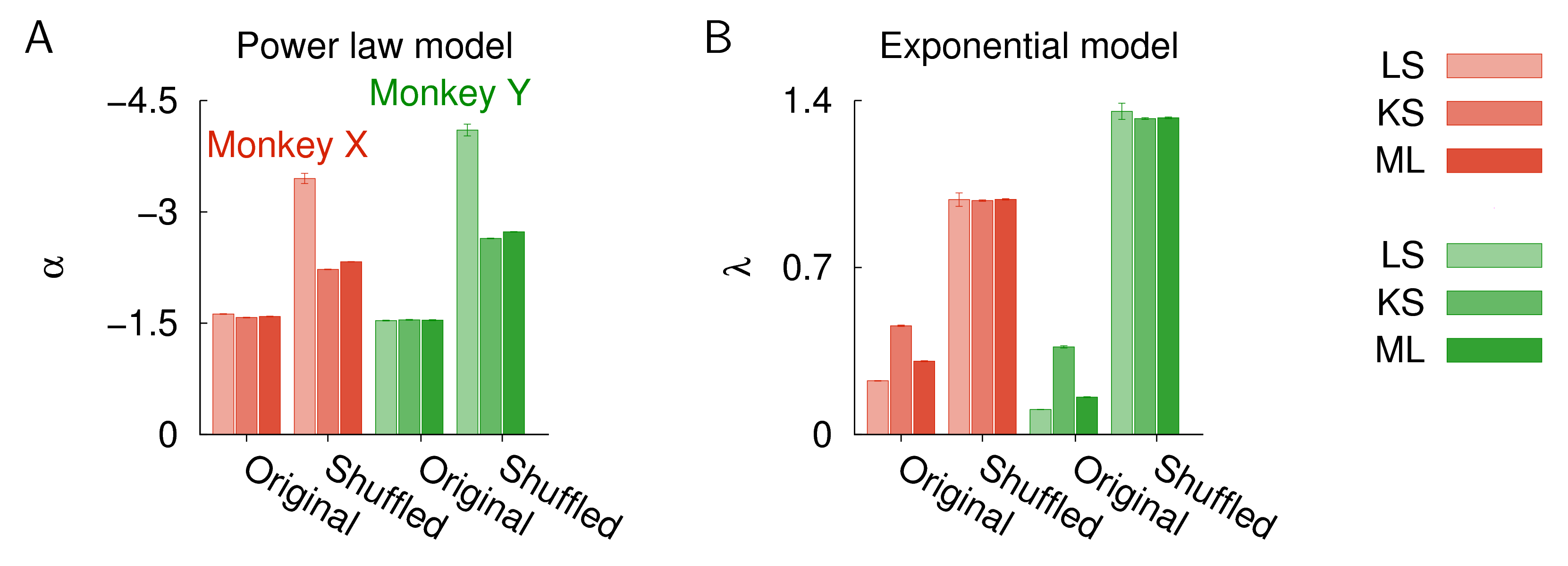

Supplement: Figure S2 — Parameter estimation for the original and the time-shuffled data in two data sets (monkey X and Y). A. Power law model with slope parameter . B. Exponential model with parameter . Three different estimation methods were compared: LS least-square estimation, KS Kolmogorov-Smirnov statistic, and ML maximum likelihood estimation. Note that all estimation methods yield similar estimates for the power law fit of the original distributions and the exponential fit of the time-shuffled distributions. However, LS estimation gave largely different values compared with KS and ML estimation when the original distribution was fitted by an exponential model, or when the power law was assumed for the time-shuffled data. The error bars denote the standard deviation for parameter estimates that were obtained by bootstrapping (200 synthetic data sets were drawn from the empirical distribution and their corresponding parameters were estimated). In some cases, the error bar is too small to distinguish. (TIF) [file pone.0019779.s005.tif]

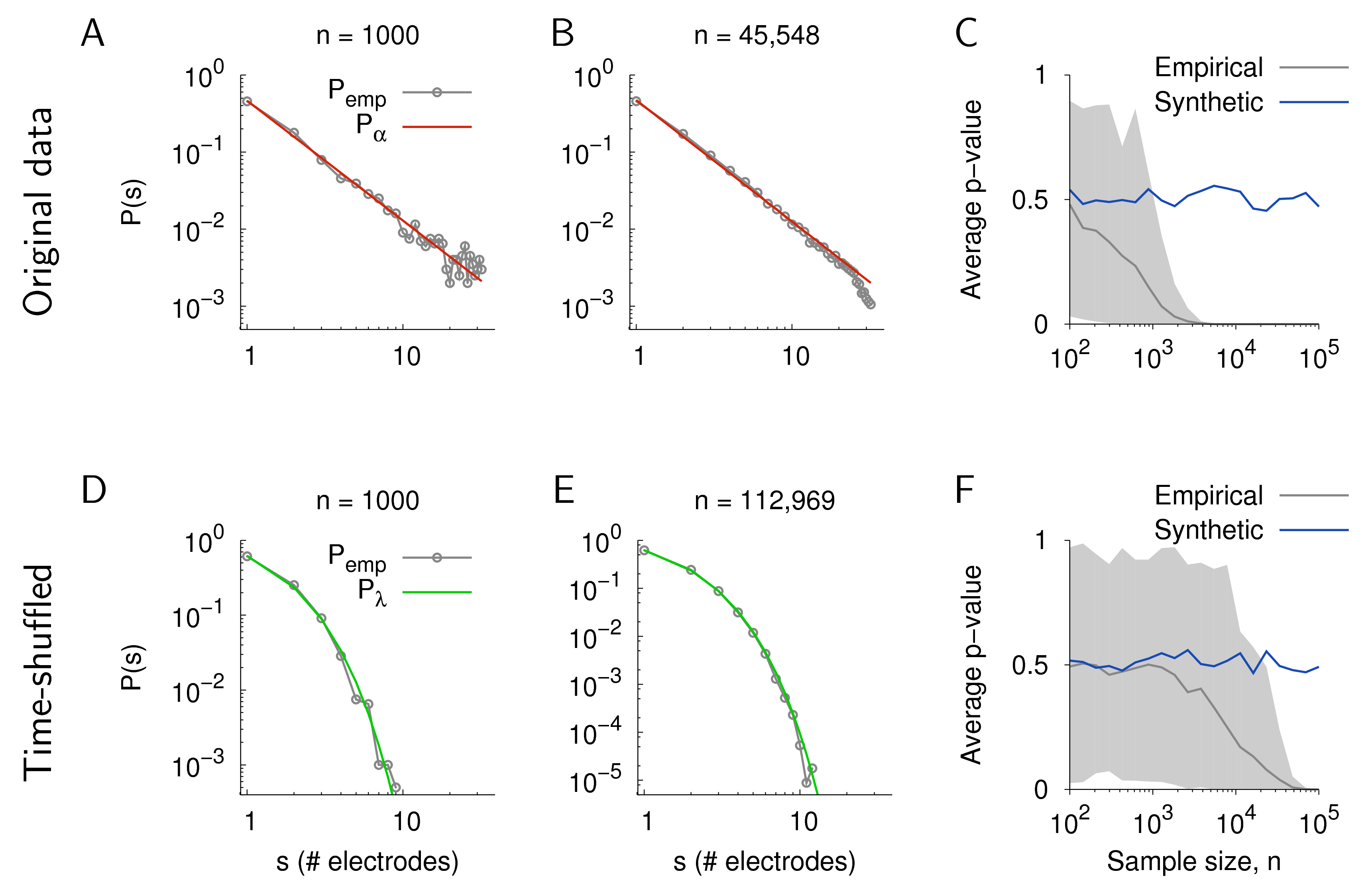

Supplement: Figure S3 — Sample size dependency of the Clauset et al. [14] goodness-of-fit evaluation. A and B. Avalanche size distribution in monkey X for a sub-set with n = 1000 and for the whole data set with n = 45,548 avalanches, respectively. Shown are the empirical PMFs, (gray), and the best-fit power law distributions, (red). C. Average p-value for different sample sizes in the empirical data (gray line). The gray area indicates the p-values between the 5th and 95th percentile. The p-value for a synthetic power law is uniformly distributed on the interval (0,1) with an average value close to 0.5 (blue line). D–F. The same for the time-shuffled data set and the exponential distribution as the model distribution (green). Note that both empirical distributions (i.e., original and rate-matched, time-shuffled data) will eventually fail against synthetic data sets given the perfect convergence of the synthetic distributions towards the model distribution for increasing n. (TIF) [file pone.0019779.s006.tif]
