# Supplementary material for: Statistical Analyses Support Power Law Distributions Found in Neuronal Avalanches
Source: PLoS One. 2011 May 26;6(5):e19779. doi: 10.1371/journal.pone.0019779 (PMC3102672; doi:10.1371/journal.pone.0019779)
Supplement: Table S1 — Comparison of the single-parameter power law with the lognormal and the gamma distribution using the LLR test for decorrelated data. (PDF) [file pone.0019779.s007.pdf]

	Power law vs. lognormal	Power law vs. gamma
In vitro	$8.0 \pm 11.8^{\text{n.s.}}$	$38.5 \pm 15.8^{\text{n.s.}}$
	$225.6 \pm 21.9^{***}$	$371.1 \pm 40.4^{***}$
	$202.8 \pm 20.2^{***}$	$436.4 \pm 26.1^{***}$
	$74.5 \pm 9.5^{***}$	$136.2 \pm 14.7^{***}$
	$69.3 \pm 11.6^{***}$	$159.2 \pm 16.8^{***}$
	$83.6 \pm 12.5^{***}$	$190.9 \pm 22.5^{***}$
	$-3.2 \pm 11.1^{\text{n.s.}}$	$-5.4 \pm 15.4^{\text{n.s.}}$
In vivo (anesthesia)	$-4.7 \pm 7.4^{\text{n.s.}}$	$1.2 \pm 10.0^{\text{n.s.}}$
	$116.8 \pm 8.3^{***}$	$186.0 \pm 14.3^{***}$
	$16.8 \pm 8.2^{\text{n.s.}}$	$32.8 \pm 12.7^{\text{n.s.}}$
	$98.6 \pm 12.1^{***}$	$156.8 \pm 16.7^{***}$
	$41.7 \pm 5.4^{***}$	$64.4 \pm 7.0^{***}$
	$40.8 \pm 12.8^{\text{n.s.}}$	$75.5 \pm 17.8^{**}$
	$10.8 \pm 11.4^{\text{n.s.}}$	$40.2 \pm 16.0^{\text{n.s.}}$
In vivo (monkey X)	$21.1 \pm 16.0^{\text{n.s.}}$	$69.4 \pm 23.1^{\text{n.s.}}$
In vivo (monkey Y)	$73.7 \pm 6.8^{***}$	$172.8 \pm 11.0^{***}$

LLR values for the comparison of the single-parameter power law with the lognormal and the gamma distribution for all data sets (i.e., 7 data sets recorded in vitro, 7 in vivo under anesthesia, and 2 in vivo in awake monkeys). Reported values are the mean±SD for decorrelated sub-sets of the data; the chosen avalanche lags were 4–26 (in vitro), 5–14 (in vivo, anesthesia), and 10 (monkey X and Y). p-values (Eq. 18, main text) and statistical significance are reported for the sub-set that had the largest p-value; $^{**}p < 0.001$, $^{***}p < 0.0001$, n.s. not significant ($p \geq 0.01$).
